# Dataset regarding the mechanical properties of roads unbound treated with synthetic fluid based on isoalkane and tall oil

**DOI:** 10.1016/j.dib.2021.107758

**Published:** 2021-12-24

**Authors:** Diego Maria Barbieri, Baowen Lou, Robert Jason Dyke, Hao Chen, Fusong Wang, Billy Connor, Inge Hoff

**Affiliations:** a Norwegian University of Science and Technology, Department of Civil and Environmental Engineering, Høgskoleringen 7A, Trondheim, Trøndelag 7491, Norway; b Chang'an University, School of Highway, Nan Er Huan Road (Mid-section), Xi'an, Shaanxi 710064, China; c Oslo Metropolitan University, Department of Civil Engineering and Energy Technology, Pilestredet 35, Oslo 0166 Norway; d State Key Laboratory of Silicate Materials for Architectures, Wuhan University of Technology, Luoshi road 122, Wuhan, Hubei 430070, China; e College of Engineering and Mines, University of Alaska Fairbanks, Tanan Loop 1760, Fairbanks, Alaska 99709, United States

**Keywords:** Stabilized roads unbound, Isoalkane, Tall oil pitch, Repeated load triaxial test, Freeze-thaw cycles, Rolling bottle test

## Abstract

The dataset revolves around the laboratory testing of an innovative additive technology for possible stabilization of unbound courses in road pavements. The product is a synthetic fluid based on isoalkane and tall oil pitch. Two test types are performed. Repeated load triaxial tests evaluate the elastic stiffness and the deformation properties of both untreated and treated aggregates. Moreover, some specimens are also tested before and after being subjected to freezing-thawing actions. A modified version of the rolling bottle test appraises the integrity with stripping loss on loose aggregates covered by the additive. Considering the necessity for road maintenance and rehabilitation worldwide, experimental data dealing with the stabilization potential of an innovative synthetic fluid stabilizer can be relevant for several road stakeholders.

**Specifications Table**

**Table:** 

Subject	Civil and Structural Engineering
Specific subject area	Stabilized roads unbound, Isoalkane, Tall oil pitch, Repeated load triaxial test, Freeze-thaw cycles, Rolling bottle test
Type of data	Table Image
How data were acquired	The data were collected by performing the following laboratory tests: Repeated Load Triaxial Test (RLTT) and modified version of Rolling Bottle Test (RBT).
Data format	Raw
Parameters for data collection	The two following laboratory tests were performed: Repeated Load Triaxial Tests (RLTTs) and modified version of Rolling Bottle Tests (RBTs). The stabilization effect related to a synthetic fluid based on isoalkane and tall oil pitch was evaluated. Six Linear Variable Differential Transducers (LVDTs) measured the vertical and horizontal deformations during RLTTs. The integrity with stripping loss was assessed by weighing each dried sample before and after RBTs.
Description of data collection	Repeated Load Triaxial Tests (RLTTs) and Rolling Bottle Tests (RBTs) were accomplished as for EN 13286-7 and EN 12697-11, respectively. 8 RLTTs samples were created and tested: 2 samples were untreated, while 6 samples were treated with different percentages of synthetic fluid and water; furthermore, 2 of these samples were also tested dried before and after being subjected to 10 Freeze-Thaw (FT) cycles. Linear Variable Differential Transducers (LVDTs) assessed the deformation of the samples inside the cyclic triaxial chamber. 84 RBT samples of loose aggregates were investigated in terms of integrity with stripping loss for fourteen rotation time intervals ranging from 1 hour to 24 hours.
Data source location	The research activities took place at the Department of Civil and Environmental Engineering, Norwegian University of Science and Technology (NTNU), Høgskoleringen 7A, Trondheim 7491, Norway. Aggregates came from a local quarry in Vassfjell, Heimdal, Norway. The synthetic fluid was provided by industrial producer (see Acknowledgments section).
Data accessibility	Dataset is uploaded on Mendeley Data Repository name: Mechanical properties of roads unbound treated with synthetic fluid based on isoalkane and tall oil Data identification number: DOI: 10.17632/x2s5mcwzdy.1 Direct URL to data: https://data.mendeley.com/datasets/x2s5mcwzdy/1
Related research article	D. M. Barbieri, B. Lou, R. J. Dyke, H. Chen, F. Wang, B. Connor, I. Hoff. Mechanical Properties of Roads Unbound Treated with Synthetic Fluid Based on Isoalkane and Tall Oil, Transportation Geotechnics. https://doi.org/10.1016/j.trgeo.2021.100701.

## Value of the Data

• Considering the high global amount of unbound layers belonging to road infrastructures that need maintenance and rehabilitation, the dataset characterizes an innovative synthetic fluid technology based on isoalkane and tall oil pitch for road stabilization.
• The dataset can be beneficial to all the road stakeholders (engineers, researchers, entrepreneurs, agencies, …) that are interested in exploring the potential of new innovative technologies for road stabilization.
• The behaviour of aggregates for road construction stabilized with an innovative synthetic fluid technology according to different application percentages can be appraised. Moreover, it is possible to analyse the data according to several regression models.

## 1 Data Description

The dataset is created during a laboratory testing campaign focusing on a Synthetic Fluid (SF) technology based on isoalkane and tall oil pitch for road stabilization [1]. The application of SF product on road aggregates represents an innovative approach to road maintenance and rehabilitation [2], [3], [4]. The investigation encompasses two kinds of test, namely Repeated Load Triaxial Test (RLTT) and a modified version of the Rolling Bottle Test (RBT), and the dataset comprises raw data and photos of the samples (https://data.mendeley.com/datasets/x2s5mcwzdy/1). The experimental campaign encompasses both untreated (Unbound Granular Material, UGM) and treated aggregates.

### 1.1 Repeated Load Triaxial Test

The folder “Data of Repeated Load Triaxial Test” contains the data derived from Repeated Load Triaxial Tests (RLTTs) and is organized in 6 subfolders corresponding to as many testing conditions as detailed in Table 1, which reports on the SF amount and water content. For each specified condition two parallel samples (indicated as “01” and “02”) were tested and the following information are reported for each specimen: one spreadsheet with raw data (.xlsx) and two pictures (.jpg).

**Table 1:** Subfolders names and corresponding testing conditions for RLTT.

Numbering	Subfolder name	SF (%, mass)	water (%, mass)
01	UGM	0	0
02	SF-1 low percentage	1.5	1
02	SF-2 medium percentage	2.5	1
02	SF-3 high percentage	4.5	1
03	after FT	1.5	0
03	before FT	1.5	0

The structure according to which all the spreadsheets are arranged has the same logic [5]. As a RLTT comprises five loading sequences, five sheets contain the corresponding experimental data and are named as “Sequence 1”, “Sequence 2”, “Sequence 3”, “Sequence 4”, “Sequence 5”; in turn, every loading sequence comprises six loading steps, which are indicated in column A. Columns B, C, D and E collect information regarding time *t*, temperature *T*, deviatoric pulse number and frequency *f*, respectively. Columns F and G specify the values of the dynamic part (*σ_d,dyn_*) and the static part (*σ_d,st_*) of the deviatoric stress *σ_d_* they are subject to. Similarly, columns H and I contain information regarding of the dynamic part (*σ_t,dyn_*) and the static part (*σ_t,st_*) of the triaxial stress *σ_t_*. The deformations of each sample are appraised by means of six Linear Variable Displacement Transformers (LVDTs), which measure the axial elastic components (*ε_a,el,01_, ε_a,el,02_, ε_a,el,03_* in columns J, L, N), axial plastic components (*ε_a,pl,01_, ε_a,pl,02_, ε_a,pl,03_* in columns K, M, O), radial elastic components (*ε_r,el,01_, ε_r,el,02_, ε_r,el,03_* in columns P, R, T) and radial plastic components (*ε_r,pl,01_, ε_r,pl,02_, ε_r,pl,03_* in columns Q, S, U).

**Fig. 1:**
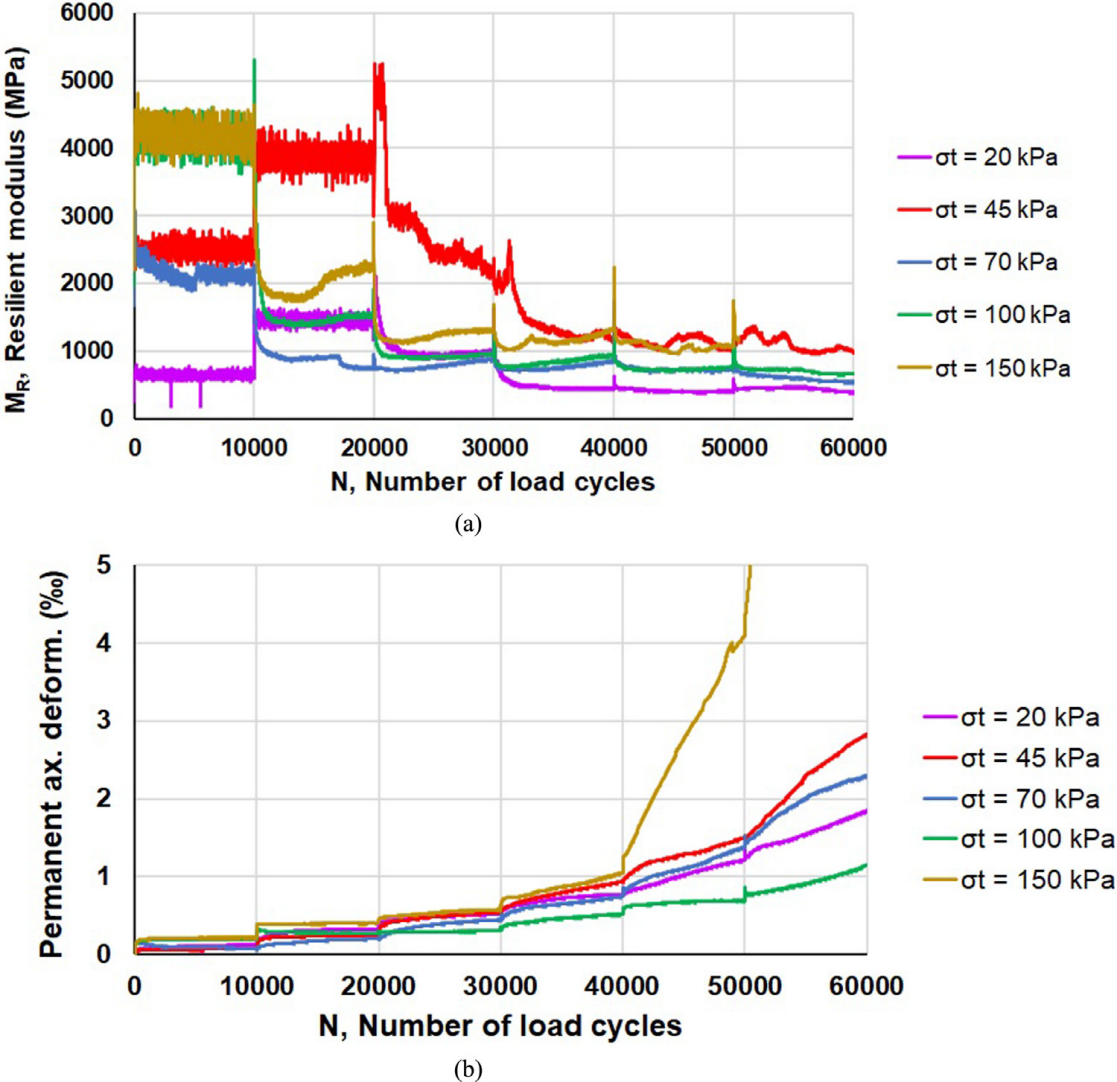
Resilient modulus *M_R_* (a) and axial plastic deformation (b) for sample 01 stabilized according to SF-1 percentage (“Spec. SF-1 01”).

Resilient modulus *M_R_* (defined in the next section) and the resistance against permanent deformation are the two main mechanical parameters that can be assessed by means of RLTTs. By way of example, the trend values of *M_R_* and axial plastic deformation for sample 01 treated according to SF-1 percentage (“Spec. SF-1 01”) are reported in Figs. 1a and 1b, respectively, as a function of load cycles number *N*. The five RLTT loading sequences correspond to as many colours in Fig. 1. Similar representations can be made for all the other RLTTs samples; furthermore, the data trend can be further analysed according to the several regression models available in literature [6,7].

### 1.2 Modified Version of Rolling Bottle Test

The folder “Data of modified version of Rolling Bottle Test” contains the file “Weight of RBT specimens.xlsx” and the folder “RBT sample pictures”. The former item specifies the weight of all the 84 RBT samples for three important conditions: dried with no SF, dried after SF application and dried after RBTs performed according to fourteen rotation time intervals (1 h, 2 h, 3 h, 4 h, 5 h, 6 h, 8 h, 10 h, 12 h, 14h, 16 h, 20 h and 24 h, reported in columns B, M). 42 samples are coated by the synthetic fluid (3% in mass) and 42 samples are not. Based on the measured weights, it is possible to quantify the parameter mass loss *ML_RBT_* as defined in the next section. Each rotation time comprises the test of three parallel samples, the pictures of which are sequentially named from “m1” to “m42”. These photos represent a supplemental visual information to observe the integrity with stripping loss and are available in .jpg format inside the folder “RBT sample pictures”, which is arranged in four subfolders “01 UGM after”, “01 UGM before”, “02 SF after”, “02 SF before”.

## 2 Experimental Design, Materials and Methods

The rock aggregates come from a local quarry located in Vassfjell, Heimdal, Norway, while the SF road stabilization technology is obtained from an industrial producer [2], [3], [4]. The research campaign took place at the Department of Civil and Environmental Engineering (Norwegian University of Science and Technology, Trondheim, Norway). The motivation of the investigation is connected with the global need to improve the maintenance and rehabilitation of road pavements [8,9]; this is particularly relevant when it comes to ensuring efficient construction and stabilization of road unbound layers [10,11].

**Fig. 2:**
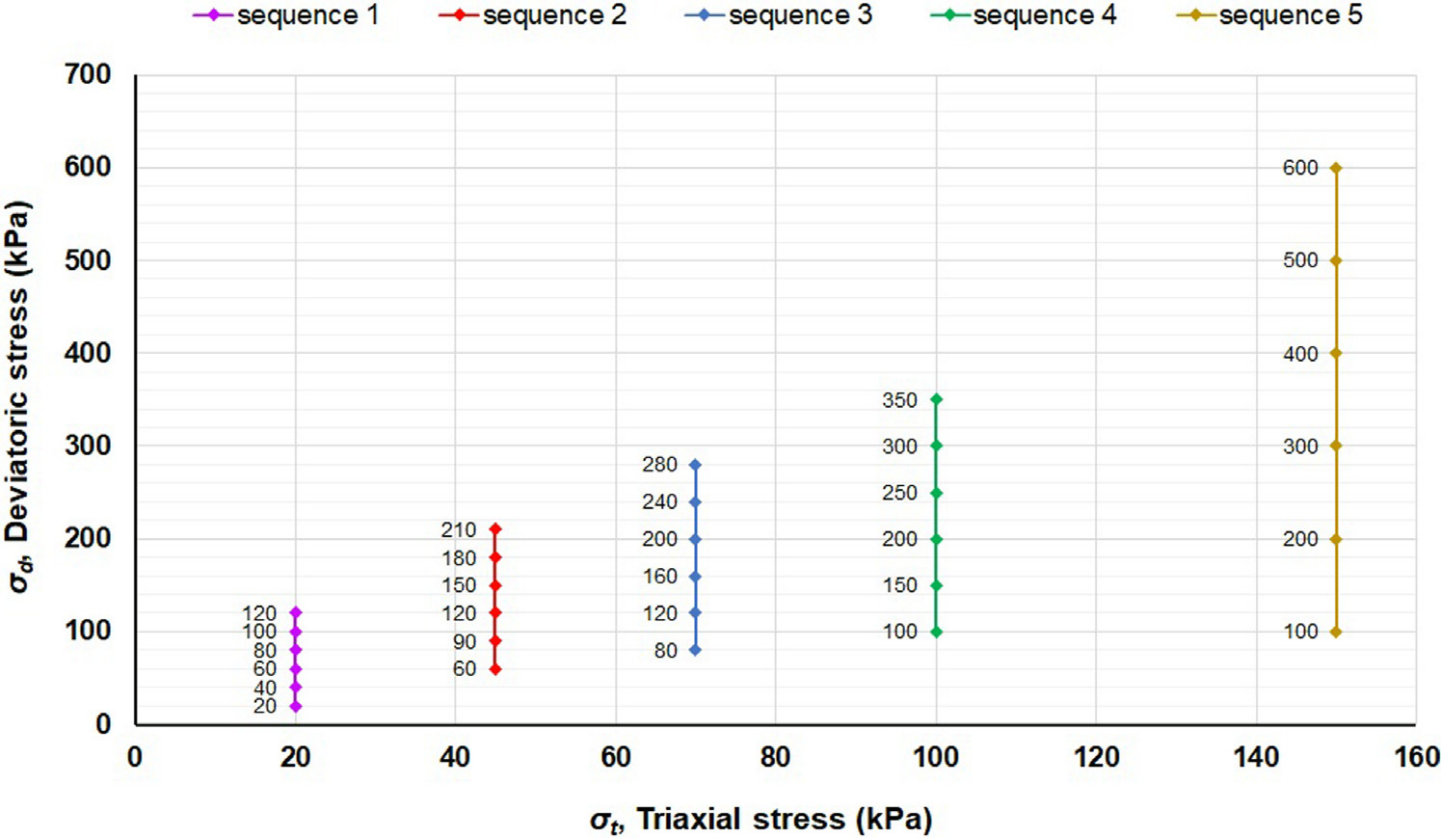
Values of triaxial stress *σ_t_* and deviatoric stress *σ_d_* defining MSL SL RLTT.

Following the specifications of CEN standard “13286-7 Cyclic load triaxial test for unbound mixtures”, each RLTT was accomplished according to the Multi-Stage Low Stress Level (MS LSL) and comprised thirty loading sequences [12], where each of them corresponded to a combination of triaxial stress *σ_t_* and deviatoric stress *σ_d_* as reported in Fig. 2. The latter one is applied according to a sinusoidal path with 10 000 pulses. For a constant value of *σ_t_* and a dynamic deviatoric stress *Δσ_d,dyn_*, the resilient modulus *M_R_* is

(1) $$M_{R}=\frac{\Delta \sigma_{d,dyn}}{\varepsilon_{a,el}},$$

with *ε_a,el_* the average axial resilient strain evaluated by the three axial LVDTs.

The RBT aims at estimating the adhesion between the bitumen covering loose aggregates and the aggregates themselves after exposure to mechanical stirring actions at room temperature [13]. According to the CEN standard “12697-11 Determination of the affinity between aggregate and bitumen”, the evaluation is performed visually, thus leading to possible unprecise outcomes. In light of this, a modified version of RBT was performed pivoting on the dry weight of the aggregates measured before (*M_1_*) and after (*M_2_*) testing. Furthermore, the standard RBT was generalized in this research as SF instead of bitumen was selected in the testing campaign and fourteen time intervals of rotations were considered (1 h, 2 h, 3 h, 4 h, 5 h, 6 h, 8 h, 10 h, 12 h, 14h, 16 h, 20 h and 24 h) instead of the two ones (6 h and 24 h) defined by the code. The mass loss *ML_RBT_* is

(2) $$ML_{RBT}=\frac{M_{1}-M_{2}}{M_{1}},$$

and can be expressed as a percentage.

## CRediT Author Statement

**Diego Maria Barbieri:** Conceptualization, Methodology, Software, Validation, Formal analysis, Investigation, Resources, Data curation, Writing – original draft, Visualization, Project administration; **Baowen Lou:** Conceptualization, Methodology, Software, Validation, Formal analysis, Investigation, Resources, Data curation, Writing - Original Draft; **Robert Jason Dyke:** Conceptualization, Methodology, Formal analysis, Investigation, Resources, Data curation, Writing - Review & Editing; **Hao Chen:** Investigation, Resources, Writing - Review & Editing, Visualization; **Fusong Wang:** Writing - Review & Editing, Visualization; **Billy Connor:** Methodology, Writing - Review & Editing, Visualization, Supervision; **Inge Hoff:** Conceptualization, Methodology, Writing - Review & Editing, Visualization, Supervision, Project administration, Funding acquisition

## Declaration of Competing Interest

This work was supported by Norwegian Public Roads Administration (VegDim project, grant number 605377) and by Research Council of Norway (HERMES project, grant number 299538).
